# Supplementary material for: Anxiety and associated factors among Ethiopian health professionals at early stage of COVID-19 pandemic in Ethiopia
Source: PLoS One. 2021 Jun 8;16(6):e0252664. doi: 10.1371/journal.pone.0252664 (PMC8186809; doi:10.1371/journal.pone.0252664)
Supplement: Attachment — Submitted filename: November 2020 comments and suggestions.pdf [file pone.0252664.s002.pdf]

Anxiety and Associated Factors among Ethiopian Health Professionals at Early Stage of COVID-19 Pandemic in Ethiopia

This manuscript is important as it assesses anxiety and associated factors among health professionals during COVID-19 which is crucial for developing an intervention plan that will address the problem. However, the introduction, methods, result, and discussion parts of the manuscript need more improvement. Also, the focuses of this study is not clear because the authors sometimes talk about depression the other time about anxiety in the introduction, method, and discussion part. So, this manuscript confuses its reader in its current status. Moreover, there are many grammatical errors that need to be edited by a language expert or a native language speaker.

Abstract

Line 44: "Developing the outcome" is not clear for readers. Try to make the term you use as simple as possible to reach all audiences.

Line 53: It would be better if you leave reporting 95% CI.

Line 54: What do you mean when you say "a large number of patients"? Do you mean treating many patients? It looks confusing.

Line 58-68: what are the implications of your findings?

Main document

Introduction

Line 67: Either delete this sentence or make it clear for readers.

Line 72 and 73: It would be better to delete depression and focus on the topic of your study.

Line 65-86: Less focus was given to anxiety. I was expecting detailed information regarding COVID-19 related anxiety but found very few sentences related to anxiety among health professionals. My recommendation is to make more literature reviews and come up with a strong introduction.

Material and Methods

Line 88-92: Information about study area is missed.

Line 90: Delete the sampling technique and report it under the topic of "sample size determination and sampling technique".

Line 95: It would be better if you report the specific social media you have used to collect data.

Line 93-96: The inclusion and exclusion criteria are not reported well.

Line 98-102: The sampling technique is not clearly mentioned, and the reason why the authors used the snowball technique is not reported. If the authors have used social media, I do not think the snowball sampling technique is the appropriate method, but your justification is important.

Line 104-111. There are varieties of questionnaires to assess anxiety but there is no clear information regarding why you selected GAD-7. Also, the specificity, sensitivity, and validity of this questionnaire in the study area and other places are not mentioned.

Line 110-111: Use reference regarding the cut-off.

Line 116: If you have analyzed the data for anxiety and depression why you did not change the topic of your study?

Result

Line 131-132, table 1: What do you mean "other health professionals"?

Discussion

Line 160-161: it is a repetition of what you wrote in the introduction. So, try to rewrite this part.

Line 166 -1167: It is better if you mention the place or countries where the previous studies were done including whether it was conducted among a similar population or not.

Line 167-168: What do you mean about the difference in the study population? Also, you were mentioning depression several times including here. So, what you have assessed in this study is not clear, is that depression or anxiety?

Line 171: it is better to leave comparing your study against population-based studies.

Line 197-201: It would be better to find other more convincing explanations regarding why females have higher anxiety during this pandemic.

Line 207-208: Why your finding is different from the study conducted in China. It is better if you discuss the differences.
